# Supplementary material for: The microbiome biomarkers of pregnant women’s vaginal area predict preterm prelabor rupture in Western China
Source: Front Cell Infect Microbiol. 2024 Oct 31;14:1471027. doi: 10.3389/fcimb.2024.1471027 (PMC11560878; doi:10.3389/fcimb.2024.1471027)
Supplement: Supplementary file 1 [file DataSheet1.zip › compare_1/Community/Pieplot/species/sample/P18.species.pieplot.pdf]

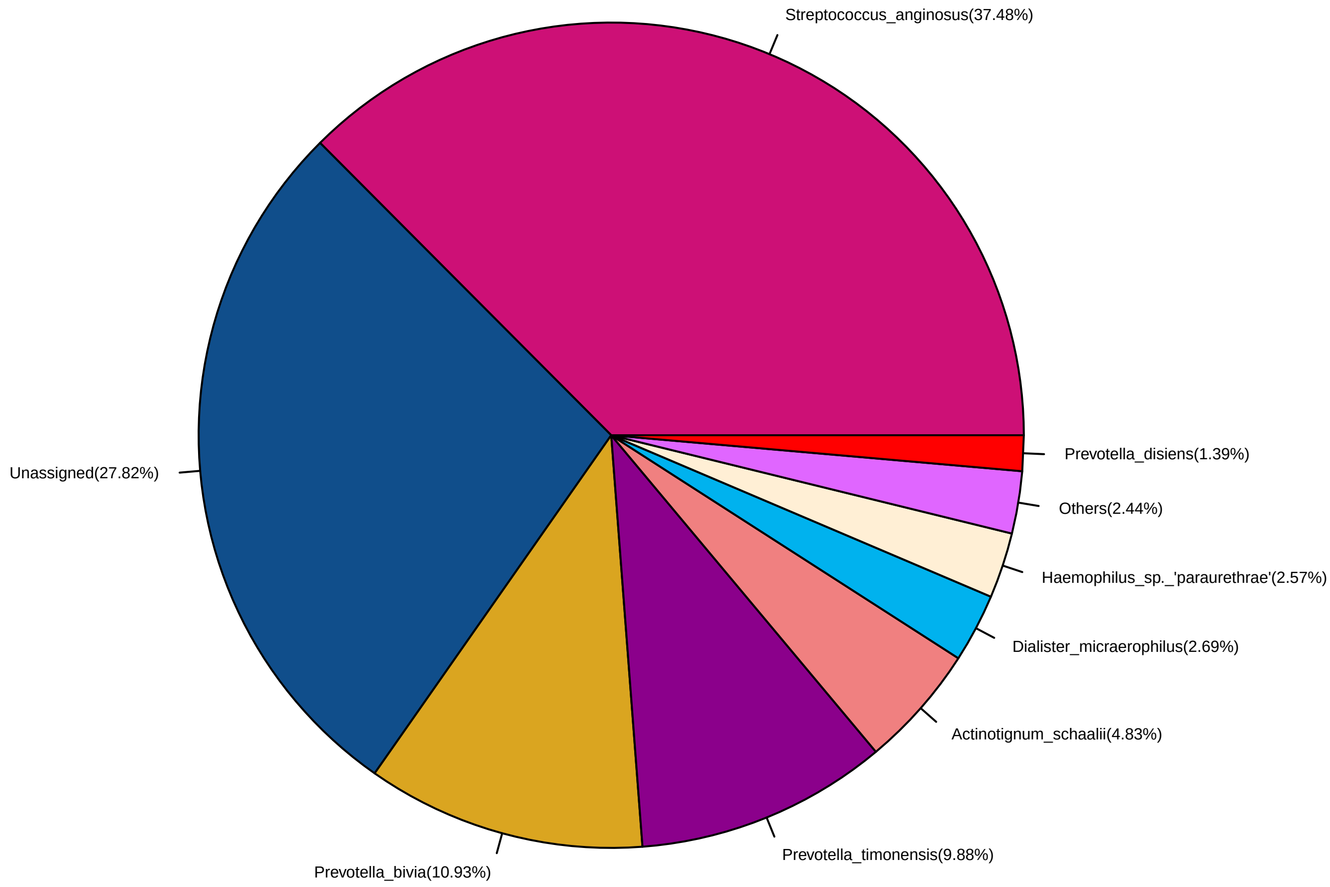
Streptococcus_anginosus(37.48%)
Unassigned(27.82%)
Prevotella_disiens(1.39%)
Others(2.44%)
Haemophilus_sp._'paraurethrae'(2.57%)
Dialister_micraerophilus(2.69%)
Actinotignum_schaalii(4.83%)
Prevotella_timonensis(9.88%)
Prevotella_bivia(10.93%)
